# Microbiota-Derived Corisin Is Elevated in Early Cervical Neoplasia and Drives Pathogenic Cellular Programs

**DOI:** 10.3390/cells15151358

**Published:** 2026-07-28

**Authors:** Naoki Watashige, Michiko Kubo-Kaneda, Marie Makino, Maya Kato, Kota Okamoto, Tsuyoshi Matsumoto, Saki Kotaka, Masaaki Toda, Corina N. D’Alessandro-Gabazza, Isaac Cann, Esteban C. Gabazza, Taro Yasuma, Kenta Yoshida, Eiji Kondo

**Affiliations:** 1 Department of Obstetrics and Gynecology, Faculty and Graduate School of Medicine, Mie University Hospital, Mie University, Tsu 514-8507, Mie, Japan; 2 Department of Immunology, Faculty and Graduate School of Medicine, Mie University, Edobashi 2-174, Tsu 514-8507, Mie, Japan; 3 Department of Animal Science, University of Illinois Urbana-Champaign, Urbana, IL 61801, USA; 4 Carl R. Woese Institute for Genomic Biology, University of Illinois Urbana-Champaign, Urbana, IL 61801, USA

**Keywords:** microbiota, corisin, cervical cancer, HeLa cells

## Abstract

**Highlights:**

**What are the main findings?**

- Circulating corisin levels were significantly elevated in patients with cervical intraepithelial neoplasia and cervical cancer, with the highest levels observed in CIN3 and cervical squamous cell carcinoma.
- Corisin was detected within cervical carcinoma tissues and induced mitochondrial accumulation, cell-cycle perturbation, apoptosis, p21 expression, and epithelial–mesenchymal transition-like changes in HeLa cells.

**What are the implications of the main findings?**

- Corisin may represent a novel microbiota-associated factor involved in the pathogenesis of cervical neoplasia.
- The presence of corisin in the cervical tumor microenvironment and its pathogenic effects on cervical cancer-derived cells support further investigation of its potential pathogenic role in cervical cancer.

**Abstract:**

Cervical cancer remains a major global health challenge and a leading cause of gynecological cancer-related mortality, particularly in developing countries. Although persistent human papillomavirus infection is the primary driver of cervical carcinogenesis, host factors such as immune dysregulation and microbiome dysbiosis may contribute to disease progression. Corisin is a microbiota-derived peptide implicated in epithelial injury and fibrosis, but its role in cervical neoplasia is unknown. To investigate its potential involvement, circulating corisin levels were measured in 27 women with cervical intraepithelial neoplasia (CIN) or cervical cancer and compared with those in 15 healthy women. Corisin localization in cervical carcinoma tissues was examined by immunohistochemistry, and its biological effects were evaluated in HeLa cells. Circulating corisin levels were significantly elevated in patients with CIN and cervical cancer, with the highest levels observed in CIN3 and cervical squamous cell carcinoma. Corisin was detected within cervical carcinoma tissues in intracellular and extracellular compartments adjacent to tumor cells. In HeLa cells, corisin accumulated in mitochondria, impaired cell-cycle progression, induced apoptosis, increased p21 expression, and promoted epithelial–mesenchymal transition-like morphological changes. These findings suggest that corisin is elevated from the early stages of cervical neoplasia, is present within the cervical tumor microenvironment, and may contribute to pathogenic cellular processes associated with cervical cancer progression.

## 1. Introduction

Cervical cancer is the fourth most common malignancy among women worldwide and remains a major global health burden, particularly in low- and middle-income countries [1,2,3]. Persistent infection with high-risk human papillomavirus (HPV) is the principal etiological factor for cervical cancer development [4]. However, although HPV infection is highly prevalent, only a small proportion of infected individuals ultimately develop cervical intraepithelial neoplasia (CIN) or invasive cervical carcinoma, indicating that additional viral, host, and environmental cofactors are necessary for malignant transformation [5,6,7]. Among these cofactors, increasing attention has recently focused on dysbiosis of the female lower reproductive tract microbiome and its contribution to persistent HPV infection, chronic inflammation, and cervical carcinogenesis [8,9].

Dysbiosis of the cervicovaginal microbiome disrupts local immune homeostasis and epithelial defense mechanisms by reducing the production of protective factors, including antimicrobial proteins, secretory leukocyte protease inhibitor, and lactic acid, and by increasing inflammatory signaling and oxidative stress [10,11,12,13,14,15]. This altered microenvironment has been associated with persistent HPV infection, increased severity of cervical dysplasia, and progression to cervical cancer [8,16]. Emerging evidence further suggests that, in addition to microbial dysbiosis itself, bacterial-derived molecules and peptides may actively participate in tumor initiation and progression by modulating epithelial signaling, inflammation, immune responses, and stromal remodeling [17,18,19,20,21,22]. Several studies have identified bacterial communities in tumor tissues, including those from cervical cancer, indicating that bacterial products may directly influence the tumor microenvironment [17,18,21].

Corisin, a microbiota-derived peptide generated through proteolytic processing of bacterial transglycosylases, has recently emerged as a novel host-interacting microbial effector identified in several commensal and pathogenic bacteria, including *Staphylococcus nepalensis*, *S. cohnii*, *S. xylosus*, and strains of *Listeria monocytogenes* and *Mycobacteroides abscessus* [23,24]. Corisin is a 19-amino-acid peptide (IVMPESSGNPNAVNPAGYR) that penetrates cells, accumulates in mitochondria, and induces apoptosis through the intrinsic mitochondrial pathway [23,24,25]. Corisin-like peptides, which differ from canonical corisin by only one or two amino acids and are encoded within transglycosylases produced by other bacterial species, including *S. haemolyticus*, also exhibit potent proapoptotic activity [24]. Beyond apoptosis, corisin has been shown to promote inflammatory cytokine production, epithelial senescence, epithelial–mesenchymal transition, and activation of coagulation pathways [24,26,27,28]. Consistent with these pleiotropic effects, corisin contributes to the pathogenesis of acute lung injury, pulmonary fibrosis, and chronic kidney injury, underscoring its role as a multifaceted mediator of epithelial injury, inflammation, and tissue remodeling [24,26,27,28]. Given that persistent epithelial injury, chronic inflammation, cellular plasticity, and stromal remodeling are key features of tumor progression, these biological properties suggest that corisin may also contribute to carcinogenesis and modulate the tumor microenvironment in microbiome-associated malignancies, including cervical cancer.

In light of accumulating evidence supporting the involvement of bacteria and bacteria-derived peptides in the tumor microenvironment, we hypothesized that corisin may be present in cervical cancer tissue and elevated in the systemic circulation of patients with cervical neoplasia. To test this hypothesis, we evaluated circulating corisin levels in patients with cervical intraepithelial neoplasia and cervical cancer and examined the presence of corisin within cervical cancer tissue. In addition, to explore the biological significance of elevated corisin levels, we investigated the effects of corisin on a cervical cancer-derived epithelial cell line.

## 2. Materials and Methods

### 2.1. Study Population

This preliminary study enrolled 8 patients diagnosed with cervical squamous cell cancer, 2 with cervical adenocarcinoma, 16 with cervical intraepithelial neoplasia (CIN), and 1 with adenocarcinoma in situ who consulted at or were referred to our institution from September 2023 through August 2024 (Table S1). The inclusion criteria were non-pregnant women diagnosed with cervical cancer or CIN who provided informed consent. The exclusion criteria included women with malignancies other than cervical cancer and those with systemic inflammatory diseases. We collected clinical, demographic, and laboratory data from electronic medical records, including information on underlying diseases. The diagnosis of CIN was made according to the National Comprehensive Cancer Network (NCCN) Clinical Practice Guidelines in Oncology for Cervical Cancer, and clinical staging was determined based on the International Federation of Gynecology and Obstetrics (FIGO) 2018 classification [29,30,31]. HPV genotyping data were available for 25 of the 27 patients. HPV DNA was detected in 21 patients (84% of those tested), whereas four patients tested negative. Samples for HPV testing were unavailable from two patients. The detected genotypes included HPV16, HPV18, HPV31, HPV33, HPV52, HPV56, HPV58, and HPV68, with multiple HPV genotypes detected in five patients (Supplementary Table S1). Corisin levels measured in blood samples from 15 healthy female volunteers were used as controls. The study protocol received approval from the Ethical Committee for Clinical Investigation at Mie University (approval number: H22023-165; date of approval: 28 August 2023). Written informed consent has been obtained from all participants.

### 2.2. Human Cervical Cancer Tissue Sections

Formalin-fixed, paraffin-embedded human tissue sections were obtained from OriGene Technologies, Inc. (Rockville, MD, USA), including specimens from a healthy control, a patient with cervical squamous cell carcinoma (stage T1a1N0M0), and another patient with cervical adenocarcinoma (stage T1aN0M0).

### 2.3. Laboratory Analysis

Patient-derived blood samples were submitted to the Clinical Laboratory of Mie University Hospital for routine hematological and biochemical assessments. Analyses included measurements of white blood cell and platelet counts, total protein, albumin, hemoglobin, aspartate aminotransferase, alanine aminotransferase, electrolytes, creatinine, and blood urea nitrogen, performed according to standardized clinical protocols. Circulating concentrations of interleukin-8 (IL-8), C-X-C motif chemokine ligand 1 (CXCL1), and p21 were determined using commercially available enzyme-linked immunosorbent assay kits (R&D Systems, Minneapolis, MN, USA) in accordance with the manufacturers’ instructions. Plasma corisin levels were quantified using an in-house enzyme-linked immunosorbent assay, as previously described [24]. It is noteworthy that this corisin ELISA system recognizes both canonical corisin and corisin-like peptides that differ from corisin by one or two amino acids.

### 2.4. Cell Culture and Corisin Stimulation

HeLa cells were maintained in Dulbecco’s Modified Eagle Medium (DMEM) supplemented with 10% fetal bovine serum, 2 mM L-glutamine, 100 IU/mL penicillin, and 100 μg/mL streptomycin, and cultured at 37 °C in a humidified atmosphere containing 5% CO_2_. For stimulation experiments, cells were grown to subconfluence, serum-starved overnight, and then exposed to corisin diluted in medium containing 0.5% recombinant human albumin (rhAlb) at final concentrations of 20 or 40 μg/mL for 48 h. In selected experiments, cells were treated with 40 μg/mL corisin in the presence of a neutralizing anti-corisin monoclonal antibody (mAb) (clone 21A) for 48 h. Corisin was used at concentrations of 20 and 40 μg/mL based on our previous studies showing reproducible biological effects of the peptide within this concentration range [24,25]. These concentrations were selected to investigate corisin-induced cellular responses and their concentration dependence and were not intended to directly reproduce the circulating corisin concentrations measured in patients. The specificity of the in-house-generated anti-corisin monoclonal antibody has been previously validated by epitope mapping and functional neutralization assays [24].

### 2.5. Synthesis and Labeling of Peptides

Corisin is a chemically synthesized, linear 19-amino acid peptide with the sequence IVMPESSGNPNAVNPAGYR. Corisin and a scrambled control peptide (NRVYNGPAASPVSEGMPIN) were synthesized by PEPTIDE Inc. (Osaka, Japan). For fluorescence imaging experiments, FITC-conjugated derivatives of both peptides (FITC-IVMPESSGNPNAVNPAGYR and FITC-NRVYNGPAASPVSEGMPIN) were synthesized by the same manufacturer during peptide synthesis. Both FITC-labeled peptides were supplied as trifluoroacetate (TFA) salts. The identity of each peptide was confirmed by electrospray ionization mass spectrometry (ESI-MS), and peptide purity, as determined by HPLC, was >99% for FITC–corisin and 98.4% for the FITC-scrambled peptide.

### 2.6. Immunocytochemical Analysis of FITC–Corisin Localization in HeLa Cells

HeLa cells were seeded at a density of 1 × 10^4^ cells/well in 24-well culture plates and cultured in DMEM supplemented with 10% FBS. After 48 h, the culture medium was replaced with DMEM containing 1% FBS. Cells were then incubated with 20 μg/mL fluorescein isothiocyanate (FITC)-labeled corisin or FITC-labeled scrambled peptide diluted in 0.5% recombinant human albumin (rhAlb) for 4 h at 37 °C. Mitochondria were stained with 50 nM MitoTracker Red CMXRos for 30 min at 37 °C. After staining, cells were washed with phosphate-buffered saline (PBS), fixed with 4% paraformaldehyde in PBS for 20 min at room temperature, and washed twice with PBS. Nuclei were counterstained with 1 μg/mL 4′,6-diamidino-2-phenylindole (DAPI) in PBS, followed by two additional PBS washes. Fluorescence images were acquired using an IX71 fluorescence microscope (Olympus Corporation, Tokyo, Japan). FITC fluorescence was detected using the WIB filter set (excitation: 460–490 nm; emission: 510LP), MitoTracker Red using the WIY filter set (excitation: 545–580 nm; emission: 610LP), and DAPI using the WU filter set (excitation: 330–385 nm; emission: 420LP).

### 2.7. Immunohistochemical Staining

Immunohistochemical staining was performed at MorphoTechnology Corporation (Sapporo, Japan) following standard protocols. The primary antibodies used included an in-house anti-corisin monoclonal antibody (clone A21; 1:300), as well as commercially available antibodies against p21 (cat. no. sc-6246; 1:50; Santa Cruz Biotechnology, Dallas, TX, USA) and p53 (cat. no. ab131442; 1:400; Abcam, Cambridge, MA, USA).

### 2.8. Immunofluorescence Staining for p21

HeLa cells were cultured in 24-well plates and stimulated with corisin (0, 20, or 40 μg/mL dissolved in 0.5% rhAlb) in the presence or absence of 200 μg/mL anti-corisin monoclonal antibody (21A). Prior to stimulation, the culture medium was replaced with DMEM supplemented with 1% FBS. After treatment, cells were washed twice with PBS without calcium and magnesium [PBS(−)] and fixed with 4% paraformaldehyde in PBS(−) for 20 min at room temperature. Cells were then washed twice with PBS(−) and permeabilized/blocked in PBS(−) containing 0.2% Triton X-100 and 1% bovine serum albumin (BSA) for 30 min at room temperature.

After removal of the blocking solution, cells were incubated overnight at 4 °C with rabbit monoclonal anti-p21 antibody (1:400 dilution; Cell Signaling Technology, Danvers, MA, USA; #2947) diluted in antibody dilution buffer (PBS containing 1% BSA and 0.2% Triton X-100). Following two washes with PBS(−), cells were incubated for 1 h at room temperature in the dark with either chicken anti-rabbit IgG Alexa Fluor 594-conjugated secondary antibody (1:500 dilution; Invitrogen, Waltham, MA, USA; A-21442) or goat anti-rabbit IgG FITC-conjugated secondary antibody (1:100 dilution; Santa Cruz Biotechnology, Dallas, TX, USA; sc-2012). Cells were then washed twice with PBS(−) and counterstained with DAPI (1 μg/mL in PBS(−)) for 5 min at room temperature. After two additional PBS(−) washes, fluorescence images were acquired using a fluorescence microscope (Olympus Corporation, Tokyo, Japan).

### 2.9. Evaluation of Cell Apoptosis

HeLa cells were seeded in 12-well plates and cultured until subconfluent. After washing, the cells were incubated for 48 h in medium containing 1% FBS and corisin at 20 or 40 µg/mL. A group of cells was treated with 40 µg/mL corisin in the presence of an anti-corisin mAb. Vehicle-treated cells served as controls. Apoptosis was assessed by flow cytometry using an FITC Annexin V Apoptosis Detection Kit with propidium iodide (PI) (BioLegend, San Diego, CA, USA), according to the manufacturer’s instructions. Fluorescence signals were analyzed using an FACScan flow cytometer (BD Biosciences, Oxford, UK).

### 2.10. Cell Cycle Analysis

HeLa cells were seeded in 12-well plates at a density of 4 × 10^5^ cells/well and cultured until confluent. The culture medium was then replaced with DMEM containing 1% FBS. Cells were treated with corisin (0, 20, or 40 μg/mL) diluted in 0.5% rhAlb in the presence or absence of 200 μg/mL anti-corisin mAb. After 24 h of stimulation, cells were harvested, washed with PBS, and transferred to 1.5 mL tubes. Cells were resuspended in 50 μL PBS by vortexing, then 1 mL of cold 75% ethanol was added dropwise while vortexing. Cells were fixed at −20 °C for 30 min and subsequently washed twice with PBS. Fixed cells were incubated with 20 μg/mL RNase A in PBS and then stained with 50 μg/mL propidium iodide (PI) in PBS. DNA content was analyzed by flow cytometry to determine cell cycle distribution.

### 2.11. Cell Count Assay

HeLa cells were seeded into 24-well plates at a density of 1 × 10^5^ cells per well. After overnight incubation, the cells were stimulated with corisin (0, 20, or 40 μg/mL) in the presence or absence of 200 μg/mL anti-corisin monoclonal antibody (clone 21A). Immediately before stimulation (0 h), a set of cells was harvested by trypsinization, washed with PBS, transferred to 1.5 mL microcentrifuge tubes, and resuspended in 50 μL PBS. The number of viable cells was determined using a hemocytometer. After 24 h of incubation, the remaining cells were harvested and processed in the same manner, and cell numbers were again determined using a hemocytometer. Four independent wells were analyzed for each experimental condition (*n* = 4).

### 2.12. Evaluation of Nuclear Morphology

Following stimulation, cells were fixed and stained with DAPI to visualize nuclear architecture. Digital images were acquired, and nuclear morphometric parameters, including nuclear area, perimeter, circularity, and roundness, were quantitatively analyzed using ImageJ version 1.54t (National Institutes of Health, Bethesda, MD, USA).

### 2.13. Evaluation of Senescence-Associated β-Galactosidase Activity

Senescence-associated β-galactosidase (SA-β-Gal) activity was assessed using X-Gal (Sigma-Aldrich; St. Louis, MO, USA) and a commercial Cellular Senescence Detection Kit (SPiDER-βGal; Dojindo, Osaka, Japan), according to the manufacturer’s instructions. Stained cells were examined using an Olympus BX50 microscope equipped with an Olympus DP70 digital camera (Olympus Corporation, Tokyo, Japan). Quantitative image analysis was performed using ImageJ version 1.54t (National Institutes of Health, Bethesda, MD, USA).

### 2.14. Evaluation of Epithelial–Mesenchymal Transition

HeLa cells were cultured for 48 h in medium containing 0.5% recombinant human albumin alone (control), corisin (20 or 40 μg/mL), or corisin (40 μg/mL) in combination with anti-corisin neutralizing monoclonal antibody. After stimulation, cells were fixed with 4% paraformaldehyde, stained with Phalloidin-iFluor™ 488 to visualize F-actin, and counterstained with DAPI to visualize nuclei. Fluorescence images were acquired under identical exposure settings using a fluorescence microscope. Quantitative analysis of F-actin fluorescence was performed using ImageJ software (NIH). Briefly, fluorescence intensity was measured in randomly selected microscopic fields after background subtraction and normalized to the number of DAPI-positive nuclei in each field. At least five independent microscopic fields were analyzed per experimental condition. In addition, expression of epithelial–mesenchymal transition (EMT)-associated genes, including vimentin (VIM), fibronectin-1 (FN1), alpha-smooth muscle actin (ACTA2/αSMA), cadherin-1 (CDH1), and transforming growth factor-β1 (TGFB1) was evaluated by RT-PCR after corisin stimulation.

### 2.15. Gene Expression Analysis

HeLa cells were cultured in 12-well plates and washed with calcium- and magnesium-free PBS. Total RNA was extracted using Sepasol-RNA I Super G reagent (Nacalai Tesque, Kyoto, Japan) according to the manufacturer’s instructions. cDNA was synthesized from 10 μg of total RNA using Moloney murine leukemia virus reverse transcriptase (TOYOBO, Osaka, Japan) and oligo(dT) primers. Real-time quantitative PCR (qPCR) was performed using the gene-specific primer sets detailed in Supplementary Table S2. Amplification was conducted using a StepOne Real-Time PCR System and PowerUp™ SYBR™ Green Master Mix (Applied Biosystems/Thermo Fisher Scientific, Waltham, MA, USA), according to the manufacturer’s instructions. Relative expression levels were normalized to hypoxanthine-guanine phosphoribosyltransferase 1 (HPRT1) as the housekeeping gene.

### 2.16. Statistical Analysis

Data are presented as mean ± standard deviation (SD). Normality of data distribution was assessed using the Kolmogorov–Smirnov and Shapiro–Wilk tests. Comparisons among three or more groups were performed using one-way analysis of variance (ANOVA), followed by Holm–Šídák post hoc testing. Statistical analyses were conducted using GraphPad Prism version 10 (GraphPad Software, San Diego, CA, USA). A two-tailed *p*-value < 0.05 was considered statistically significant.

## 3. Results

### 3.1. High Circulating Levels of Corisin from the Early Stages of Cervical Cancer

**Figure 1:**
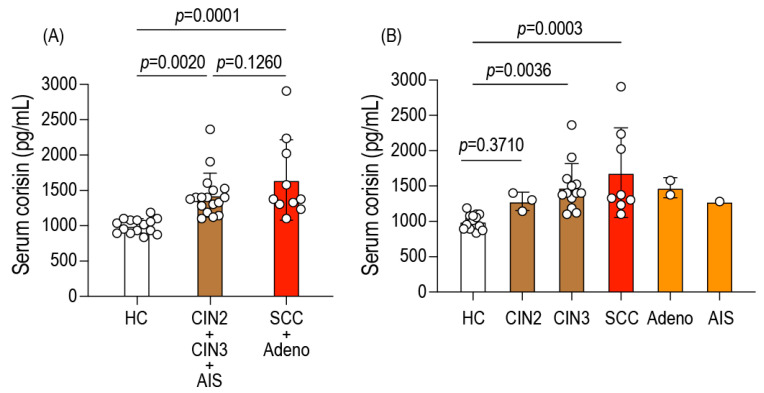
Elevated circulating corisin levels in cervical intraepithelial neoplasia and invasive cervical cancer. Serum corisin concentrations were measured by enzyme immunoassay in healthy controls and patients with cervical intraepithelial neoplasia (CIN) or cervical cancer. (**A**) Comparison of serum corisin levels among healthy controls (HC; *n* = 15), CIN+AIS (*n* = 17), and invasive cervical cancer groups (*n* = 10). (**B**) Stratified analysis of serum corisin levels in healthy controls, CIN2, CIN3, cervical squamous cell carcinoma (SCC), adenocarcinoma, and adenocarcinoma in situ (AIS). Data are presented as mean ± SD, with individual data points shown as open circles. Statistical analysis was performed using ANOVA followed by the Holm–Šídák post hoc test.

Elevated circulating levels of corisin were observed in patients diagnosed with CIN and cervical cancer, compared to a healthy control cohort (Figure 1A). In addition, serum corisin levels were significantly higher in patients with CIN3 and cervical squamous cell carcinoma than in healthy controls (Figure 1B). Although serum corisin concentrations tended to be higher in patients with cervical cancer than in those with CIN, this difference did not reach statistical significance. These findings suggest that corisin is markedly increased in the systemic circulation from the early stages of cervical carcinogenesis. However, given the relatively small sample size and the lack of significant differences in serum corisin levels between CIN and more advanced stages of the disease, it remains unclear whether circulating corisin levels increase further with disease progression.

### 3.2. Detection of Corisin Within Cervical Carcinoma Tissue

**Figure 2:**
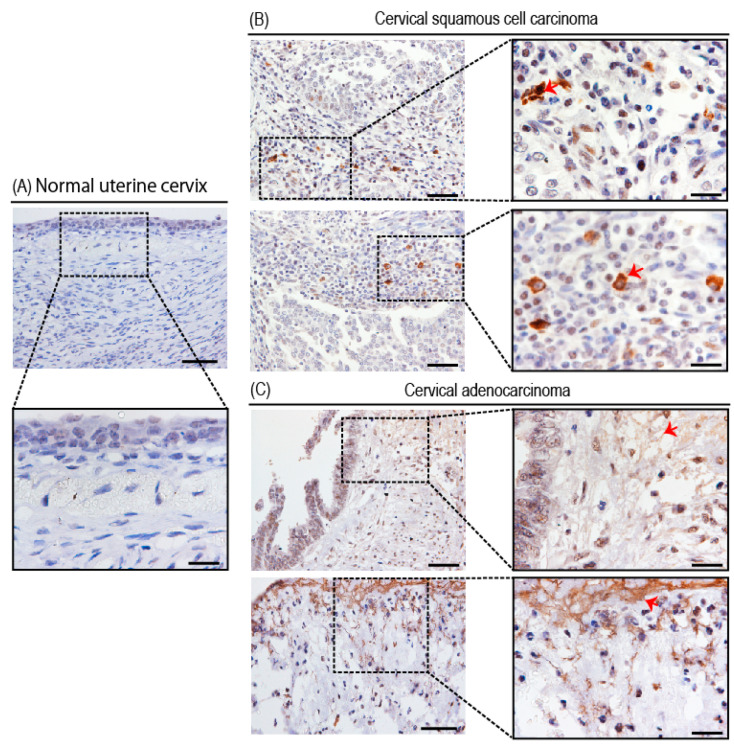
Detection of corisin in human cervical cancer tissues. (**A**) Representative immunohistochemical staining demonstrating corisin immunoreactivity in normal cervical tissue from a healthy subject. (**B**) Representative immunohistochemical staining demonstrating corisin immunoreactivity in cervical squamous cell carcinoma (SCC) tissue. (**C**) Representative immunohistochemical staining demonstrating corisin immunoreactivity in cervical adenocarcinoma tissue. Dashed boxes indicate regions shown at higher magnification in the lower panels. Red arrows denote tumor cells exhibiting positive cytoplasmic corisin staining. Immunostaining was performed using an in-house-generated anti-corisin monoclonal antibody and visualized with 3,3′-diaminobenzidine (DAB), followed by hematoxylin counterstaining. Scale bars: 50 µm (left panels) and 20 µm (right panels).

After demonstrating elevated circulating levels of corisin in patients with cervical neoplasia, we next examined whether corisin was also present within cervical cancer tissue. Immunohistochemical staining was performed using an in-house-developed anti-corisin monoclonal antibody. Corisin immunoreactivity was detected in cervical carcinoma specimens within tumor cells, as well as in the extracellular stromal compartment in close proximity to tumor cells within the tumor interstitium. Intracellular corisin immunoreactivity was observed predominantly in the cytoplasmic and perinuclear regions of tumor cells. In contrast, little to no corisin immunoreactivity was observed in cervical mucosal tissue from a healthy control (Figure 2A–C). These findings indicate that corisin is detectable within cervical cancer tissue, suggesting that cervical cancer cells may be directly exposed to corisin in the tumor microenvironment. However, because paired tumor tissue samples were not available from the patients included in the serum analysis, the present study could not determine the proportion of corisin-positive cases across CIN2, CIN3, and cervical cancer, nor assess the correlation between tissue corisin immunoreactivity and circulating corisin levels.

### 3.3. Corisin Accumulates in the Mitochondria of a Cervical Cancer-Derived Cell Line

**Figure 3:**
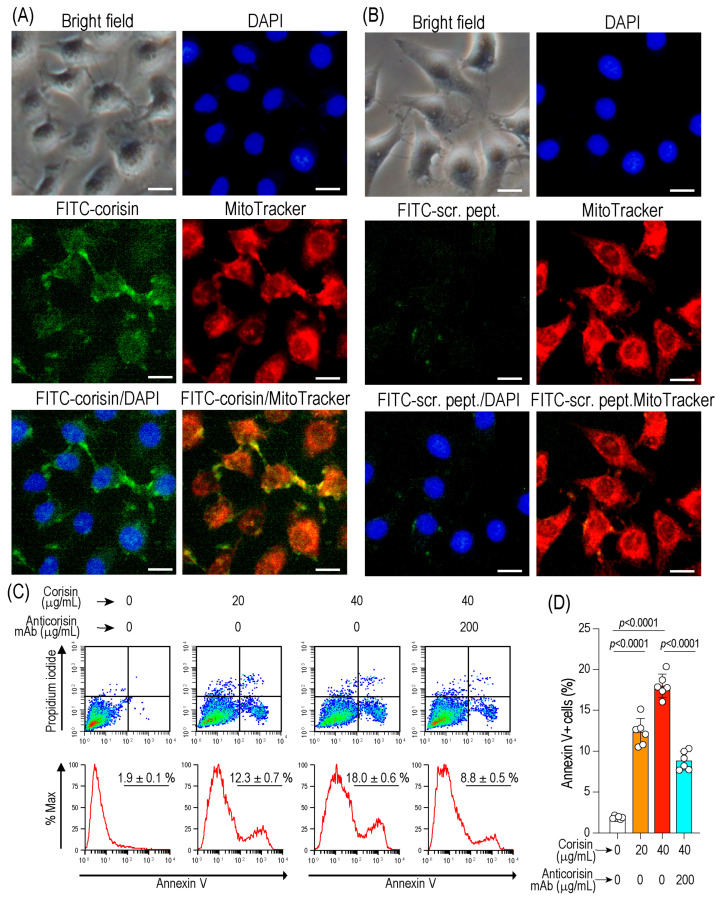
Corisin accumulates in mitochondria and induces apoptosis in HeLa cells, which is inhibited by an anti-corisin monoclonal antibody. (**A**) Representative fluorescence microscopy images showing localization of FITC-labeled corisin in mitochondria of HeLa cells. Cells were incubated with FITC-corisin (green), stained with MitoTracker (red) to visualize mitochondria, and counterstained with DAPI (blue). Scale bars indicate 10 µm. (**B**) Representative fluorescence microscopy images of HeLa cells treated with FITC-labeled scrambled peptide (FITC-scr. peptide). The scrambled peptide showed no co-localization with mitochondria. Scale bars indicate 10 µm. (**C**) Representative flow cytometric analysis of apoptosis in HeLa cells treated with corisin in the presence or absence of anti-corisin monoclonal antibody (mAb, clone 21A). Cells were treated with corisin (20 or 40 μg/mL) with or without anti-corisin mAb (200 μg/mL), followed by Annexin V/propidium iodide staining. Representative dot plots and Annexin V histograms are shown. (**D**) Quantification of Annexin V-positive cells. Each group included six replicates (*n* = 6). Data are presented as mean ± SD. Statistical analysis was performed using ANOVA followed by Holm–Šídák post hoc test. Scale bars = 10 μm.

Previous studies have demonstrated that corisin accumulates in the mitochondria of parenchymal cells in several organs [24,25]. Therefore, after detecting corisin not only in the cervical cancer microenvironment but also intracellularly within tumor cells, we next investigated whether corisin similarly accumulates in the mitochondria of cervical cancer-derived HeLa cells. Fluorescence microscopy showed that FITC-labeled corisin was taken up by HeLa cells and displayed a punctate intracellular distribution that partially overlapped with MitoTracker-positive mitochondrial structures, indicating preferential mitochondrial accumulation. In contrast, FITC-labeled scrambled peptide showed no mitochondrial co-localization (Figure 3A,B). These findings suggest that corisin preferentially accumulates in the mitochondria of HeLa cells.

### 3.4. Corisin Impairs Cell Cycle Progression and Induces Apoptosis in Cervical Cancer-Derived HeLa Cells

Single-cell transcriptomic studies have shown that exposure of normal parenchymal cells to corisin induces multiple injury-associated cellular responses, including apoptosis, senescence-associated transcriptional programs, and epithelial–mesenchymal transition [25]. We therefore examined whether corisin elicits similar biological effects in cervical cancer-derived HeLa cells. We first assessed apoptosis by flow cytometry using Annexin V/propidium iodide staining. Corisin increased the proportion of Annexin V-positive cells in a dose-dependent manner (Figure 3C,D). Importantly, co-treatment with an anti-corisin monoclonal antibody markedly attenuated corisin-induced apoptosis, supporting the specificity of this effect.

**Figure 4:**
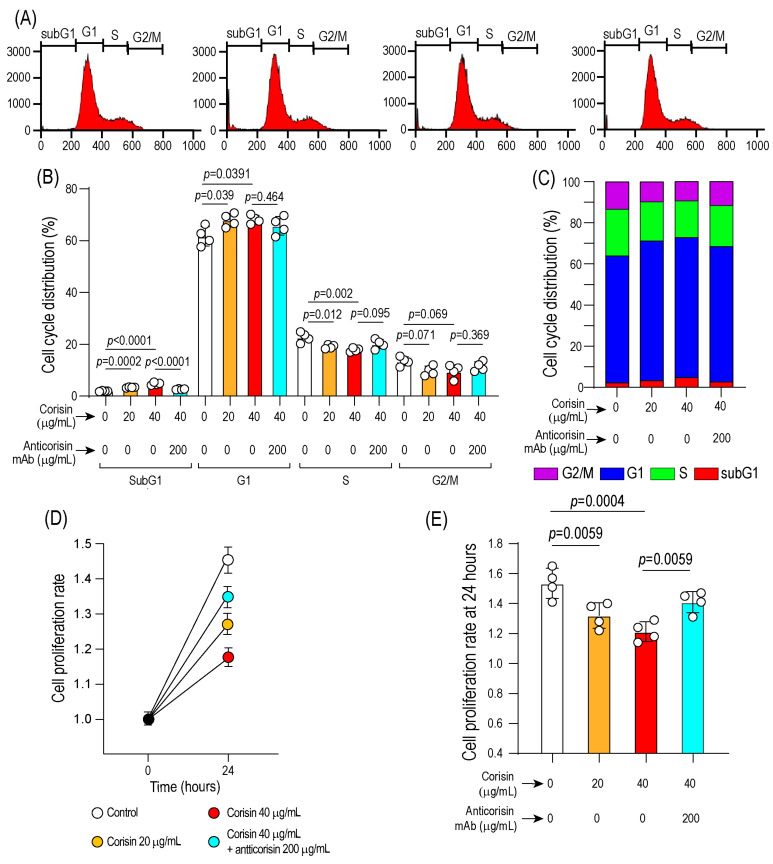
Corisin impairs cell cycle progression and suppresses proliferation in HeLa cells. (**A**–**C**), Cell cycle distribution in HeLa cells treated with corisin, with or without anti-corisin monoclonal antibody, was assessed by flow cytometry. Representative histograms are shown in (**A**), quantitative analysis of sub-G1, G1, S, and G2/M populations in (**B**), and stacked distribution of cell cycle phases in (**C**). Each experimental group included four replicates (*n* = 4). (**D**) Time-course analysis of relative HeLa cell proliferation. Cell proliferation was normalized to the value at 0 h (set to 1.0). Symbols correspond to the treatment groups shown below the graph. (**E**) Quantification of relative HeLa cell proliferation at 24 h for the treatment groups shown in panel (**D**). Each experimental group included four replicates (*n* = 4). Data are presented as the mean ± SD. Statistical analysis was performed using ANOVA followed by the Holm–Šídák post hoc test.

We next evaluated whether corisin affects cell cycle progression. Cell cycle analysis showed that corisin increased the sub-G1 and G1 populations while reducing the S-phase population (Figure 4A–C), indicating increased accumulation of apoptotic cells together with perturbation of cell cycle progression. These alterations were ameliorated by co-treatment with the anti-corisin monoclonal antibody. Consistent with these findings, corisin exposure significantly reduced the proliferation rate of HeLa cells (Figure 4D,E). Together, these results demonstrate that corisin impairs cell cycle progression and proliferation while promoting apoptosis in HeLa cells.

Because apoptotic cells can paradoxically stimulate the proliferation of neighboring cancer cells through caspase-dependent mitogenic signaling, a process known as apoptosis-induced compensatory proliferation [32], the proapoptotic activity of corisin may contribute to cervical cancer progression by promoting compensatory growth of surviving cancer cells during chronic exposure.

### 3.5. Corisin Induces p21 Expression, Senescence-Associated β-Galactosidase Activity, and SASP-Related Responses in Cervical Cancer-Derived Cells

**Figure 5:**
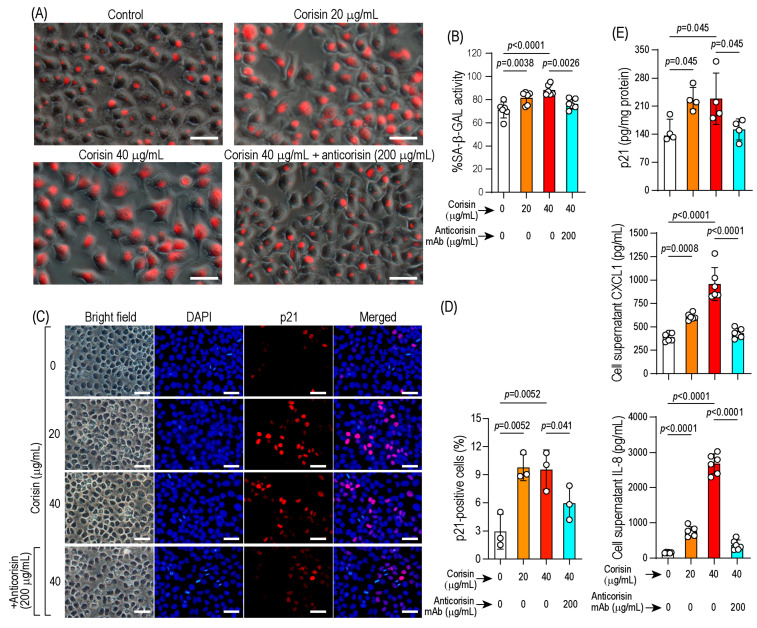
Corisin induces senescence-associated cellular responses in HeLa cells. (**A**) Representative phase-contrast images overlaid with senescence-associated β-galactosidase (SA-β-gal) signal in HeLa cells cultured under control conditions or treated with corisin (20 or 40 µg/mL), in the presence or absence of a neutralizing anti-corisin monoclonal antibody (mAb; 200 µg/mL). Scale bars indicate 20 µm. (**B**) Quantitative analysis of SA-β-gal activity. Each group included six replicates (*n* = 6). (**C**) Representative bright-field and immunofluorescence images showing p21 expression in HeLa cells after corisin stimulation. Nuclei were counterstained with DAPI (blue), and p21-positive cells are shown in red. Scale bars indicate 20 µm. (**D**) Quantification of p21-positive cells. Each group included three replicates (*n* = 3). (**E**) Quantification of intracellular p21 protein levels in cell lysates (four replicates per group; *n* = 4) and measurement of the SASP-associated factors (six replicates per group; *n* = 6), CXCL1 and IL-8, in culture supernatants. Bars represent the mean ± SD. Statistical analysis was performed using ANOVA followed by the Holm–Šídák post hoc test.

We next investigated whether corisin induces cellular responses associated with senescence in cervical cancer-derived cells. To address this question, we evaluated senescence-associated β-galactosidase (SA-β-gal) activity, p21 expression, and the production of senescence-associated secretory phenotype (SASP)-related factors. Corisin treatment significantly increased SA-β-gal activity, p21 immunoreactivity, and p21 protein levels in cell homogenates compared with untreated control cells (Figure 5A–E). Importantly, co-treatment with an anti-corisin antibody markedly attenuated the corisin-induced increases in SA-β-gal activity, p21 expression, and p21 immunoreactivity, supporting the specificity of these effects. In addition, corisin significantly upregulated the expression of the SASP-associated factors IL-8 and CXCL1, whereas their induction was substantially suppressed in the presence of the anti-corisin antibody (Figure 5E).

**Figure 6:**
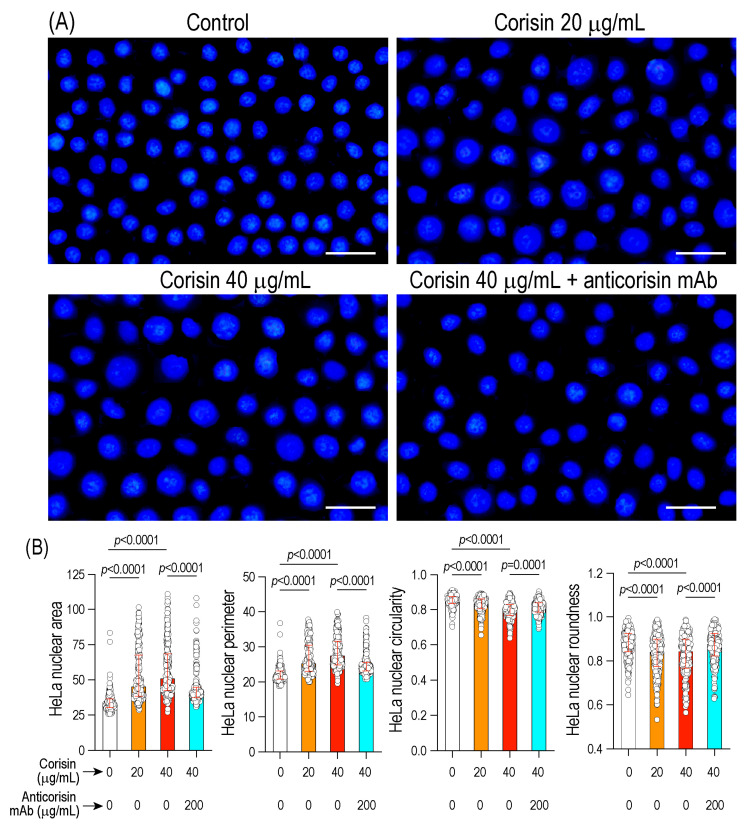
Corisin alters nuclear morphology in HeLa cells. HeLa cells were cultured under control conditions or treated with corisin (20 or 40 µg/mL), in the presence or absence of a neutralizing anti-corisin monoclonal antibody (mAb; 200 µg/mL). (**A**) Representative DAPI-stained images (blue) showing nuclear morphology under each condition. Corisin treatment was associated with increased nuclear size and altered nuclear shape, which were attenuated by concomitant treatment with the anti-corisin mAb. Scale bars indicate 20 µm. (**B**) Quantitative analyses of nuclear area, nuclear perimeter, nuclear circularity, and nuclear roundness. Scale bars indicate 20 µm. Four replicates were included in each group. The number of nuclei evaluated was 218 in the control group (corisin, 0 μg/mL), 214 in the corisin 20 μg/mL group, 215 in the corisin 40 μg/mL group, and 218 in the corisin 40 μg/mL plus anti-corisin antibody group. Bars represent the mean ± SD, with individual nuclei shown as open circles. Statistical analysis was performed using ANOVA followed by the Holm–Šídák post hoc test.

Because alterations in nuclear morphology have been reported in cells exhibiting senescence-associated phenotypes, we further examined nuclear morphology in HeLa cells following corisin treatment. Exposure to corisin increased nuclear area and perimeter and reduced nuclear circularity and roundness compared with untreated control cells (Figure 6A,B).

Together, these findings demonstrate that corisin induces multiple senescence-associated cellular features, including increased SA-β-gal activity, p21 expression, production of SASP-related factors, and nuclear morphological alterations. Given the emerging role of senescence-associated cellular programs in tumor progression and invasiveness [33,34,35], these observations suggest that corisin may contribute to cervical cancer progression and enhanced tumor aggressiveness.

### 3.6. Increased p21 and p53 Immunoreactivity in Cervical Carcinoma

**Figure 7:**
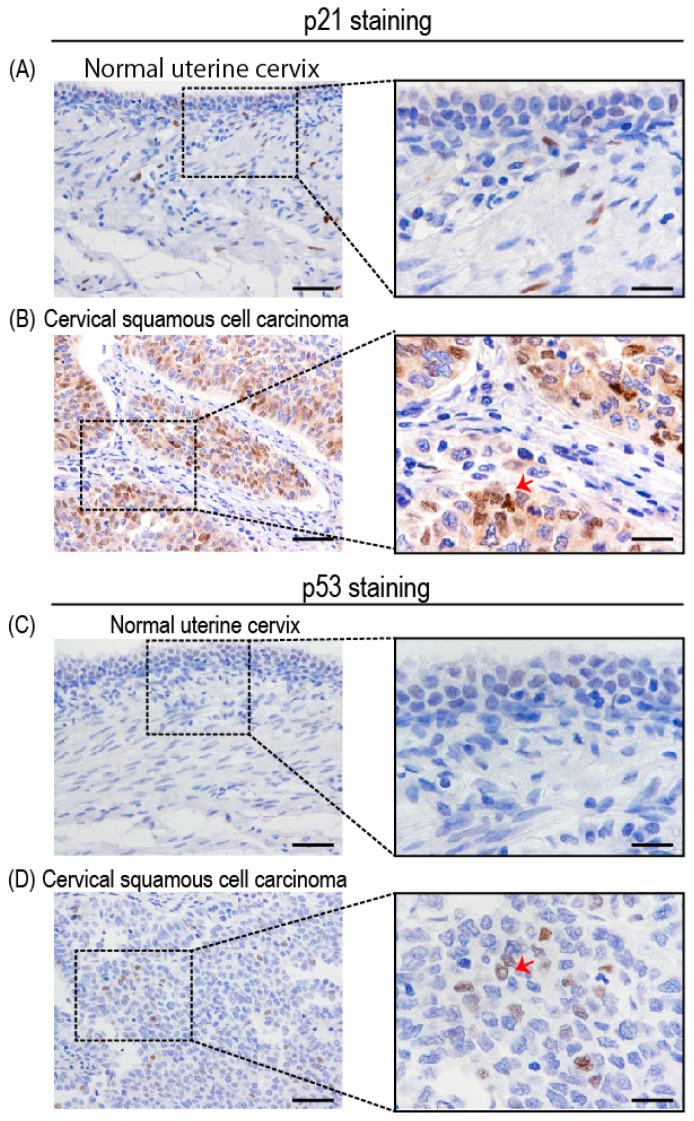
Increased p21 and p53 immunoreactivity in cervical squamous cell carcinoma. Immunohistochemical staining for p21 in normal uterine cervix (**A**) and cervical squamous cell carcinoma (**B**), and for p53 in normal uterine cervix (**C**) and cervical squamous cell carcinoma (**D**), was performed as described in the Section 2. Representative images are shown. Boxed areas in the left panels are shown at higher magnification in the corresponding right panels. Red arrows denote tumor cells exhibiting positive immunoreactivity for p21 in (**B**) or p53 in (**D**). Scale bars: 50 µm in low-magnification images and 20 µm in high-magnification images.

To evaluate the expression of stress- and cell-cycle-associated markers in vivo, immunohistochemical staining for p21 and p53 was performed in cervical carcinoma tissues. In normal uterine cervix specimens, p21 staining was weak and limited to a small number of scattered epithelial cells (Figure 7A). In contrast, cervical SCC tissue showed marked p21 immunoreactivity in tumor cells, with prominent cytoplasmic and nuclear staining patterns (Figure 7B, arrow). Similarly, p53 immunostaining was barely detectable in normal uterine cervix tissue (Figure 7C), whereas cervical SCC tissue exhibited increased p53-positive tumor cells with evident nuclear staining (Figure 7D, arrow).

These findings demonstrate increased expression of p21 and p53 in cervical SCC tissue, consistent with enhanced cellular stress responses and altered regulation of cell-cycle-associated pathways in the tumor microenvironment.

### 3.7. Corisin Promotes EMT in Cervical Cancer-Derived Cells

**Figure 8:**
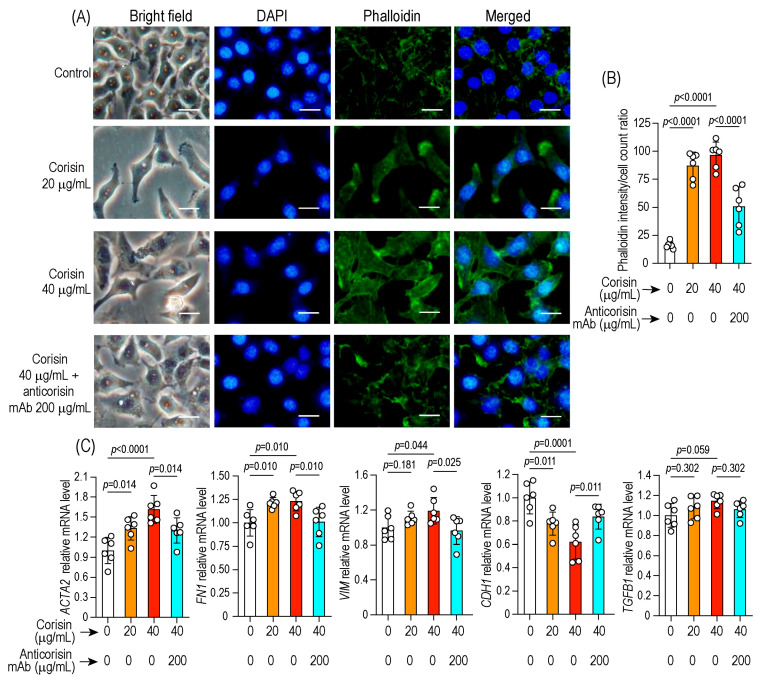
Corisin induces EMT-associated phenotypic remodeling in HeLa cells. HeLa cells were cultured under control conditions or treated with corisin (20 or 40 µg/mL), in the presence or absence of a neutralizing anti-corisin monoclonal antibody (200 µg/mL). (**A**) Representative bright-field, DAPI (nuclei, blue), phalloidin (F-actin, green), and merged images are shown. Corisin treatment was associated with marked morphological changes, including cell elongation, loss of epithelial cobblestone-like morphology, and reorganization of the F-actin cytoskeleton. These changes were attenuated by concomitant treatment with the anti-corisin monoclonal antibody. Scale bars indicate 10 µm. (**B**) Quantitative analysis of EMT-associated morphological changes. (**C**) Relative mRNA expression levels of EMT-associated genes in HeLa cells following corisin stimulation. Scale bars indicate 10 µm. Data are presented as mean ± SD with individual data points shown. Statistical analysis was performed using ANOVA followed by the Holm–Šídák post hoc test.

Epithelial–mesenchymal transition (EMT) is a key biological process implicated in tumor aggressiveness, metastatic dissemination, and therapeutic resistance in multiple cancers, including cervical cancer [36,37]. Because corisin induced cellular senescence and increased SASP-associated factors, which are known to promote EMT, we next hypothesized that corisin may drive EMT in cervical cancer-derived cells. To test this hypothesis, HeLa cells were cultured with corisin in the presence or absence of a neutralizing anti-corisin monoclonal antibody. Corisin treatment induced marked morphological alterations consistent with EMT, including acquisition of a fibroblast-like phenotype and reorganization of the F-actin cytoskeleton, as visualized by phalloidin staining, compared with untreated control cells. Notably, these EMT-associated phenotypic changes were significantly attenuated by co-treatment with the anti-corisin antibody (Figure 8A,B).

We next examined the expression of canonical EMT-related genes, including α-smooth muscle actin (ACTA2), fibronectin (FN1), vimentin (VIM), E-cadherin (CDH1), and transforming growth factor-β1 (TGFB1). Corisin significantly increased the relative mRNA expression of ACTA2, FN1, and VIM, whereas it significantly decreased CDH1 expression compared with control cells. These corisin-induced changes were significantly attenuated by co-treatment with the anti-corisin monoclonal antibody (Figure 8C). In contrast, TGFB1 expression did not differ significantly among the treatment groups.

Together, these findings indicate that corisin promotes EMT-associated phenotypic and transcriptional remodeling in cervical cancer-derived cells, suggesting that corisin may contribute to enhanced cervical cancer cell invasiveness.

## 4. Discussion

The present study demonstrates that circulating levels of the microbiota-derived peptide corisin are significantly elevated in patients with cervical neoplasia, including those with early-stage disease. Corisin was detectable within cervical carcinoma tissues and exerted biological effects on cervical cancer-derived cells by inducing mitochondrial injury, apoptosis, cell-cycle perturbation, p21 expression, senescence-associated β-galactosidase activity, and EMT-like phenotypic remodeling.

Cervicovaginal dysbiosis has increasingly been recognized as an important cofactor in the pathogenesis of cervical cancer beyond persistent HPV infection alone. Multiple studies have demonstrated that cervical intraepithelial neoplasia and cervical cancer are associated with depletion of protective *Lactobacillus* species and enrichment of anaerobic, proinflammatory bacterial communities within the vaginal microbiome [9,38,39]. In particular, an increased abundance of bacterial genera associated with epithelial injury, chronic inflammation, and tumor-promoting microenvironments, including *Gardnerella*, *Prevotella*, *Sneathia*, *Fusobacterium*, and *Stenotrophomonas*, has been reported in patients with cervical dysplasia and cervical cancer [40]. Within this context, our finding of elevated corisin levels in both systemic circulation and cervical cancer tissue suggests that dysbiosis may influence not only microbial composition but also the production and release of bioactive bacterial peptides.

Although neither the composition of the cervicovaginal microbiome nor the direct presence of corisin-producing bacteria within cervical tumor tissues was evaluated in the present study, previous microbiological analyses have identified several bacterial species capable of producing transglycosylases containing corisin or corisin-like peptide motifs, including *Staphylococcus cohnii*, *S. haemolyticus*, *S. hominis*, and *S. warneri*, within the vaginal microenvironment under both healthy and pathological conditions [41,42,43,44,45,46,47]. These observations raise the possibility that cervicovaginal dysbiosis may increase the local availability of corisin or corisin-like peptides within the cervical microenvironment. Given that corisin has been shown to induce apoptosis, cell-cycle perturbation, p21 expression, senescence-associated β-galactosidase activity, EMT-like phenotypic remodeling, and inflammatory responses, chronic local exposure to corisin may contribute to epithelial injury, stromal remodeling, and modulation of the tumor microenvironment in cervical cancer.

The precise biological origin of corisin detected in patients with cervical neoplasia and the mechanisms by which this microbiota-derived peptide reaches the cervical microenvironment remain unknown. In addition to its potential local production by members of the cervicovaginal microbiota, corisin produced at distant microbial sites, including the gut, may cross a compromised epithelial barrier, enter the systemic circulation, and subsequently reach the cervical tissues via the bloodstream. In this regard, emerging evidence suggests that the gut microbiome may influence the cervicovaginal microbial ecosystem through the gut–vagina axis via mechanisms including microbial exchange, immune modulation, endocrine regulation, and gut-derived metabolites [48]. These local and systemic routes are not mutually exclusive, and both local production and systemic delivery may contribute to corisin exposure within the cervical microenvironment. Future studies integrating paired gut and cervicovaginal microbiome analyses with measurements of circulating corisin and its localization within cervical tissues will be required to determine its biological origin and route of delivery.

Our present findings, together with previous work, further indicate that corisin elicits diverse biological responses depending on the cellular context and the degree of stress it imposes [25]. In the present study, the magnitude of corisin-induced apoptosis, proliferative inhibition, and senescence-associated changes was moderate and did not involve the entire cell population, suggesting heterogeneity in the cellular response to corisin. Nevertheless, corisin increased Annexin V positivity several-fold, reduced cell proliferation, altered cell-cycle distribution, and enhanced SA-β-gal activity, with these effects being attenuated by neutralization with an anti-corisin monoclonal antibody. These findings suggest that corisin does not induce a uniform cellular fate but rather promotes distinct pathogenic responses in different cellular subpopulations, including apoptosis, impaired proliferation, and senescence-associated phenotypic changes. Consistent with this interpretation, single-cell transcriptomic analyses have shown that corisin exposure can simultaneously induce apoptosis, senescence-associated transcriptional programs, and EMT-related or myofibroblastic differentiation within the same epithelial population [25].

The determinants of the heterogeneous outcomes induced by corisin remain unclear but may involve corisin concentration, exposure duration, mitochondrial and metabolic status, cellular stress intensity, and the efficiency of intracellular peptide uptake. In this regard, our recent data suggest that corisin may enter cells, at least in part, through the albumin receptor cubilin, potentially influencing intracellular accumulation and downstream signaling [25]. Importantly, clinical and preclinical studies have linked elevated corisin levels to tissue fibrosis, apoptosis, senescence-associated cellular responses, and EMT-related changes in the lung and kidney [24,25,27]. Intratracheal administration of corisin exacerbates pulmonary fibrosis and EMT-associated changes, whereas systemic administration promotes renal fibrosis in experimental models [23]. Collectively, these observations support a model in which corisin functions as a multifaceted mediator of epithelial injury, cellular plasticity, inflammation, and tissue remodeling, processes that may also contribute to cervical cancer progression and tumor microenvironment dynamics.

At first glance, the observation that corisin induces apoptosis and suppresses proliferation in HeLa cells appears paradoxical, given that sustained proliferative signaling and resistance to programmed cell death are fundamental hallmarks of cancer [49]. However, increasing evidence indicates that apoptosis and tumor progression are not always mutually exclusive [32,50,51,52,53]. In established tumors, persistent or repeated apoptotic injury can promote tumor evolution through apoptosis-induced compensatory proliferation, a caspase-dependent process in which dying cells stimulate the proliferation of neighboring surviving cells [32,51]. In addition, chronic epithelial injury and cell turnover create a proinflammatory tissue microenvironment that may facilitate tumor progression [49]. Thus, although corisin directly induces apoptosis in cervical cancer cells under experimental conditions, chronic exposure to this microbiota-derived peptide in vivo could paradoxically contribute to cervical carcinogenesis by promoting continuous epithelial injury, compensatory proliferation of surviving cells, and tissue remodeling. This concept is consistent with our finding of elevated corisin levels in cervical cancer tissues and serum, although the precise contribution of apoptosis-induced compensatory proliferation to cervical cancer progression remains to be established experimentally.

Several limitations should be acknowledged. First, the study included a relatively small number of participants from a single center, particularly when the patient cohort was subdivided according to disease stage. This limited sample size may have reduced the statistical power to detect differences between CIN and more advanced cervical cancer and precluded robust adjustment for potential confounding factors. It may also limit the generalizability of the findings across different populations and geographic regions. Therefore, the clinical findings should be considered exploratory and require validation in larger, multicenter cohorts with well-balanced disease-stage groups. Second, HPV status was not assessed in the healthy control cohort, HPV viral load was not measured, and the number of HPV-negative patients was too small for a sufficiently powered stratified analysis. Consequently, the present findings do not establish whether elevated circulating corisin is independent of HPV infection. Third, the mechanistic experiments were performed exclusively in HeLa cells, which are HPV18-positive and exhibit extensive chromosomal abnormalities and long-term adaptation to in vitro culture. Future studies should therefore examine the effects of corisin in multiple cervical cancer cell lines with different HPV backgrounds, as well as in non-transformed cervical epithelial cells. Fourth, the corisin concentrations used in vitro were higher than those detected in serum and may not reflect local tissue exposure. Therefore, these experiments should be considered mechanistic proof of concept, and the physiological relevance of these concentrations requires further investigation. Finally, another important limitation of this study is that although corisin induced mitochondrial dysfunction, apoptosis, p21 expression, cellular senescence, and EMT-associated phenotypic changes, the signaling pathways linking corisin exposure to these responses were not investigated. Therefore, the present findings establish cellular associations and biological effects but do not define a complete molecular mechanism. Future studies should examine candidate stress- and apoptosis-related pathways using pathway-specific inhibitors, gene silencing, and molecular activation analyses.

## 5. Conclusions

In conclusion, this study identifies corisin as a microbiota-derived peptide that is elevated during early cervical neoplasia, is present in cervical cancer tissue, and induces mitochondrial dysfunction, apoptosis, p21 expression, senescence-associated β-galactosidase activity, and EMT-associated phenotypic remodeling in cervical cancer-derived cells. These findings extend the biological effects of corisin beyond previously studied fibrotic diseases and suggest that it may contribute to the link between cervicovaginal dysbiosis, epithelial injury, and microenvironmental remodeling in cervical neoplasia. However, the present study does not delineate the intracellular signaling pathways connecting corisin exposure to these cellular responses. Further studies using pathway-specific molecular and pharmacological approaches, as well as in vivo models, are required to define the upstream microbial sources, downstream signaling mechanisms, and relevance of corisin to cervical disease progression.

## Data Availability

The raw data supporting the findings of this study are available in Zenodo at https://doi.org/10.5281/zenodo.20767792.

## Supplementary Materials

The following supporting information can be downloaded at: https://www.mdpi.com/article/10.3390/cells15151358/s1. Table S1: Clinical and laboratory profiles of the patients; Table S2: Primers used for gene expression analyses.

## Abbreviations

The following abbreviations are used in this manuscript:

AIS	adenocarcinoma in situ
CIN	cervical intraepithelial neoplasia
CXCL1	C-X-C motif chemokine ligand 1
DAPI	4′,6-diamidino-2-phenylindole
EMT	epithelial–mesenchymal transition
FIGO	International Federation of Gynecology and Obstetrics
HPV	human papillomavirus
IL-8	interleukin-8
mAb	monoclonal antibody
NCCN	National Comprehensive Cancer Network
SA-β-gal	senescence-associated β-galactosidase
