# Supplementary material for: Microbiota-Derived Corisin Is Elevated in Early Cervical Neoplasia and Drives Pathogenic Cellular Programs
Source: Cells. 2026 Jul 28;15(15):1358. doi: 10.3390/cells15151358 (PMC13463999; doi:10.3390/cells15151358)
Supplement: Supplementary file 1 [file cells-15-01358-s001.zip › cells-4418473-supplementary.pdf]

Supplementary Materials

Microbiota-Derived Corisin Is Elevated in Early Cervical Neoplasia and Drives Pathogenic Cellular Programs

Naoki Watashige, Michiko Kubo-Kaneda, Marie Makino, Maya Kato, Kota Okamoto, Tsuyoshi Matsumoto, Saki Kotaka, Masaaki Toda, Corina N D'Alessandro-Gabazza, Isaac Cann, Esteban C. Gabazza, Taro Yasuma, Kenta Yoshida, Eiji Kondo.

Table S1. Clinical and laboratory profiles of the patients

	Units	CIN	Cervical cancer	All patients	Normal range
No of patients		17	10	27	
Age (mean ± SD)	Years-old	36.9 ± 7.1	39.1 ± 7.3	38.1 ± 6.9	
BMI		22.8 ± 4.4	21.7 ± 3.4	22.4 ± 4.0	
Comorbidity				1	
Adenomyosis		1		1	
Panic disorder		1		1	
Attention-Deficit/Hyperactivity Disorder			1	1	
Diabetes mellitus, Hypertension			1	1	
Bronchial asthma		1		1	
Stage					
IIA1		1		1	
IB1		1		1	
IB2		2		2	
IB3		3		3	
ICIII		2		2	
TNM					
T2a			1	1	
T1b1			2	2	
T1b2			2	2	
T1b3			5	5	
NX			1	1	
N0			7	7	
N1			2	2	
M0			10	10	
Histopathological diagnosis					
CIN2		3		3	
CIN3		12		12	
AIS		1		1	
CIN3 + AIS		1		1	
Squamous cell carcinoma			8	8	
Adenocarcinoma			2	2	
Arterial blood pressure					
Systolic blood pressure	mmHg	112.2 ± 11.9	114.5 ± 14.9	113.7 ± 12.8	90 – 120
Diastolic blood pressure	mmHg	71.2 ± 8.4	73.0 ± 13.1	72.3 ± 10.2	60 – 80
Laboratory data					
White blood cells	x 10^3 μL	7.5 ± 2.3	11.6 ± 3.4	9.2 ± 3.4	3.3 – 8.6
Red blood cells	x 10^6 μL	3.9 ± 0.3	3.6 ± 0.4	3.8 ± 0.4	3.8 – 5.0
Hemoglobin	g/dL	11.7 ± 0.9	10.3 ± 1.4	11.2 ± 1.3	11.6 – 14.8
Hematocrit	%	35.7 ± 2.7	31.9 ± 3.7	34.2 ± 3.7	35.1 – 44.4
Platelets	x 10^4 μL	250.8 ± 61.4	249.6 ± 45.8	249.6 ± 55.8	15.8 – 34.8
Total protein	g/dL	6.6 ± 0.5	5.0 ± 0.7	6.0 ± 1.0	6.6 – 8.1
Albumin	g/dL	3.6 ± 0.2	2.8 ± 0.4	3.3 ± 0.5	4.1 – 5.1
Blood urea nitrogen	mg/dL	10.2 ± 2.4	7.9 ± 2.5	9.2 ± 2.7	8 – 20
Creatinine	mg/dL	0.5 ± 0.1	0.6 ± 0.1	0.6 ± 0.1	0.46 – 0.79
eGFR	mL/min/1.73m^2	101.7 ± 32.3	91.2 ± 18.3	96.4 ± 27.5	≥ 60
Na	mEq/L	139.4 ± 1.4	138.7 ± 2.0	139.1 ± 1.6	138 – 145
K	mEq/L	3.8 ± 0.4	4.1 ± 0.5	3.9 ± 0.4	3.6 – 4.8
Cl	mEq/L	106.2 ± 2.2	109.2 ± 1.9	107.4 ± 2.6	101 – 108
Aspartate Aminotransferase	U/L	19.4 ± 8.2	15.5 ± 7.3	18.0 ± 8.0	13 – 30
Alanine Aminotransferase	U/L	17.9 ± 14.8	10.2 ± 4.6	15.0 ± 12.6	7 – 23
Lactate Dehydrogenase	U/L	143.8 ± 52.0	111.5 ± 13.4	142.5 ± 50.7	124 – 222
Creatine Phosphokinase	U/L	87.5 ± 135.1	94.8 ± 61.7	92.9 ± 111.0	41 – 153
Human papillomavirus status					
Positive		13	8	21	
Negative		3	1	4	
Not determined		1	1	2	

CIN, Cervical Intraepithelial Neoplasia; AIS, adenocarcinoma in situ

Table S2. Primers used for gene expression analyses

Gene	Direction	Sequence (5' to 3')	Length	Tm	Reference	Position	cDNA
TGFβ1	Forward	CTAATGGTGGAAACCCACAACG	21	61.4	NM_000660	334-355	209 bp
	Reverse	TATCGCCAGGAATTGTTGCTG	23	60.7		542-522	
MKI67	Forward	AGAAGAAGTGGTGCTTCGGAA	21	61.3	NM_002417	1936-1956	202 bp
(Ki-67)	Reverse	AGTTTGCGTGGCCTGTACTAA	21	61.7		2137-2117	
FN1	Forward	CGGTGGCTGTCAGTCAAAG	19	60.7	NM_212482	125-143	130 bp
(Fibronectin)	Reverse	AAACCTCGGCTTCCTCCATAA	21	60.9		254-234	
VIM	Forward	GAGAACTTTGCCGTTGAAGC	20	58.0	NM_003380.5	1476-1495	163 bp
(Vimentin)	Reverse	GCTTCCTGTAGGTGGCAATC	20	58.6		1638-1619	
CDH1	Forward	GTATCTTCCCCGCCCTGCCAATCC	24	75.9	NM_004360.5	2512-2535	765 bp
(E-cadherin)	Reverse	GTATCTTCCCCGCCCTGCCAATCC	24	75.9		2512-2535	765 bp
ACTA2		GTGTTGCCCCTGAAGAGCAT	20	62.5	NM_001613	290-309	109 bp
(α-smooth muscle actin)		GCTGGGACATTGAAAGTCTCA	21	60.0		398-378	
HPRT1	Forward	TGACCAGTCAACAGGGGACA	20	60.7	NM_000194.3	468 - 487	189 bp
	Reverse	CGTGGGGTCCTTTTCACCAG	20	60.9		656 - 637	

TGFβ1, transforming growth factor -β1; MIKI67, marker of proliferation Ki-67; HPRT1, hypoxanthine-guanine phosphoribosyltransferase.
